# Personality associations with online vs. offline social capital and life satisfaction

**DOI:** 10.1186/s40359-024-02265-9

**Published:** 2024-12-19

**Authors:** Miao Chao, Dmitri Rozgonjuk, Jon D. Elhai, Haibo Yang, Christian Montag

**Affiliations:** 1 Key Research Base of Humanities and Social Sciences of the Ministry of Education, Academy of Psychology and Behavior, Tianjin Normal University, Tianjin, China; 2 Department of Molecular Psychology, Institute of Psychology and Education, Ulm University, Helmholtzstr. 8/1, 89081 Ulm, Germany; 3 Institute of Computer Science, University of Tartu, Tartu, Estonia; 4 Department of Psychology, and Department of Psychiatry, University of Toledo, Toledo, OH USA; 5 Faculty of Psychology, Tianjin Normal University, Tianjin, China

**Keywords:** Social capital, Personality, Life satisfaction, Big five, Extraversion, Agreeableness

## Abstract

Social capital is an important construct in diverse scientific disciplines for understanding health promotion, entrepreneurship, and economic growth. In an increasingly digitalized world, social capital can be established and used in both online and offline contexts. Previous research suggests that personality might be relevant to an understanding of individual differences in social capital. For instance, the literature suggests that extraversion is associated with more social capital. Against this background, the present study aimed to revisit social capital research, but with a broader focus on studying all Big Five Personality traits (assessed with the BFI-45) and their association with bonding (similarity-based relationships), bridging (diversity-based relationships) social capital dimensions, and well-being. Insights in social capital variables in offline and online areas were obtained via the Internet Social Capital Scale and well-being was assessed with Diener’s Satisfaction with Life scale. In particular, the study aimed to understand if personality-well-being associations would emerge with online/offline social capital being a mediator. The questionnaires were filled in by *n* = 289 German speaking participants (73 males and 216 females). The results revealed that offline social capital in the form of bridging and bonding played a significant role in mediating the relationship between both agreeableness and extraversion with life satisfaction. Online social capital was not associated with life satisfaction and was only very weakly linked to some Big Five Personality traits. In conclusion, the present study demonstrates that offline social capital is very relevant for well-being, while online social capital shows no association with self-reported well-being levels and seems to be negligible for an understanding of well-being.

**Supplementary Information:**

The online version contains supplementary material available at 10.1186/s40359-024-02265-9.

## Introduction

Social capital has been broadly defined as “a collective asset in the form of shared norms, values, beliefs, trust, networks, social relations, and institutions that facilitate cooperation and collective action for mutual benefits” (p. 480) [1]. Social capital is of high relevance for many areas in society, such as health promotion [2], entrepreneurship [3], and economic growth [4]. Social capital research in psychology has profited from the introduction of a self-report tool providing insight into individual differences of two facets of social capital named “bridging” and “bonding” [5]. “Bridging social capital” measures connecting with the outer world (outside of their existing social networks) and are therefore “bridges” between different milieus. The “bonding” facet of social capital assesses if persons can rely on social resources in their close social networks, with whom they trust and can talk.

With the rise of social media, there has been a growing interest in studying online social capital. For instance, it has been shown that informational use of the Chinese social media platform WeChat was linked to broader network capital (but only assessed within the online realm) [6]. Further research links online social capital in particular bonding to life satisfaction [7]. This is of course also of relevance for the present study investigating online and offline social capital in the context of well-being. Another study investigated the role of cognitive and structural social capital in the context of information sharing. This study did not distinguish between online and offline social capital and also the information sharing part was not divided into online vs. offline information sharing [8]. Therefore, the study is less relevant for our present content.

The study of social capital has a long history (but entered academic scrutiny only in the 1990s) [1]. Despite prior effort to investigate this concept, the question arises if online vs. offline social capital link differently to well-being variables. This could be the case, because more direct human interaction might be a more important source of well-being, in particular in times of crisis (the pandemic was illustrative in that way [9, 10]).

Personality psychologists have aimed at understanding which personality traits are linked with bridging and bonding facets of social capital. Of relevance for the present study, a recent meta-analysis found support for the “rich-get-richer hypothesis,” where extraverted people use social networking sites to enhance their social capital online, resulting in more social resources. In contrast, loneliness and social anxiety could not facilitate the accumulation of online social resources [11]. In sum, those who are characterized with greater inner urges for social belonging (extraversion) seem to profit more from social media platforms and related services. More detailed views stemming from empirical research revealed that extraversion seems to be slightly more associated with online bonding than online bridging, although effect sizes were both weak [12, 13]. In detail the work by Weiqin et al [12] showed correlations of 0.13 vs 0.07, whereas the work by Williams [13] showed correlations of 0.12 vs. 0.09. The latter work has also shown that extraversion seems to be more strongly related to offline bridging and offline bonding than to their online counterparts [13]. This research question is one we wish to revisit with the present work. In line with the observed studies, it has also been shown that higher extraversion goes along with more bridging on the social media platform LinkedIn [14]. Interestingly, that study investigated the complete Big Five model and observed that aside from higher extraversion (*r =* .20), higher agreeableness (*r =* .28) was associated with more bridging (a significant effect was also observed for neuroticism: *r =* .12). The study did not investigate offline social capital.

The present study’s first aim is to investigate the association between the Big Five personality traits and bonding and bridging facets of social capital. Both online and offline social capital settings are studied here. Against the background of the literature, we expect that extraversion is positively correlated with bonding social capital, and we also expect that extraversion plays a pivotal role in understanding bridging. As the literature suggests [13], we expect relevant personality associations to be weaker in the online context than in the offline context. As Williams’ study is more than 15 years old, it will be interesting to see if such differences can still be observed in a stronger digitalized world where social media platforms play an important role in establishing social capital [15, 16].

Finally, our study also aims to shed light on the relationship between social capital and well-being. It is well known that personality traits are linked to well-being [17], and also social capital has been linked to well-being [18]. Extraversion is well-known to be associated with greater well-being [19], and extraversion has been shown to be of particular relevance for bridging and bonding social capital [13, 20]. Therefore, we expect positive associations between these variables. As extraversion represents a rather stable trait, we expect extraversion’s positive association with the state well-being variable to be mediated by the bridging and bonding facets, because we expect establishing/being able to use one’s own social capital to be a source of well-being (and the latter to be particular true for extraverts). Given the human need for direct social interaction (e.g., Maslow’s theory) [21], we anticipate that offline bridging and offline bonding will play a significant role in the mediation model. Further investigation in the present study is exploratory.

## Method

### Participants

A total of 289 German speaking participants (73 males and 216 females; M_age_=29.26, *SD* = 10.76; range 18–70) were recruited for the present study via a larger project investigating individual differences in the context of several topics linked to digitalization in Germany (for instance a paper on technology-self-efficacy and attitudes towards AI was already published) [22]. Not only students were allowed to participate, but also people from the general population. Participants needed to be at least 18 years or older. Participants also provided insights into their objective smartphone behavior and some biological markers – yielding what are sometimes called digital biomarkers [23]. The study was advertised via various channels on and off campus.

All participants provided informed consent to participate in the study, and among others, filled in the questionnaires, as mentioned below, in the German language. This study was approved by the local ethics committee of Ulm University, Ulm, Germany.

### Measurements

#### Personality

We used the validated German version of the 45-item Big Five Personality Inventory [24] to measure personality traits. This inventory assesses five personality dimensions: extraversion, agreeableness, conscientiousness, neuroticism, and openness to experience. Participants rated their level of agreement with statements describing these traits on a 5-point scale ranging from 1 (“strongly disagree”) to 5 (“strongly agree”). The inventory consisted of 8 items for extraversion, 10 for agreeableness, 9 for conscientiousness, 8 for neuroticism, and 10 for openness to experience. Internal consistency is presented in Table 1.

#### Social capital

The Internet Social Capital Scale (ISCS) [5] was used to evaluate individuals’ perceived social capital in both online and offline contexts. The ISCS has two scales (online and offline), each with two subscales (bonding and bridging) consisting of 10 items each. Respondents rated their agreement on a 5-point scale from 1 (“completely disagree”) to 5 (“completely agree”). The scores for each subscale ranged from 10 to 50. Sample items included “When I feel lonely, there are several people online/offline I can talk to” for bonding, and “Online/Offline, I come in contact with new people all the time” for bridging. In previous studies, the ISCS showed good reliability and construct validity [25]. The German version of the scale was translated and back-translated by two native German speakers, both holding doctoral degrees in psychology.

#### Life satisfaction

The German version of the Satisfaction with Life Scale [26, 27] was used to assess an individual’s overall life satisfaction. It consists of five items, with responses rated on a Likert scale ranging from 1 (“strongly disagree”) to 7 (“strongly agree”). A sample item is “The conditions of my life are excellent.”

### Analyses

We used SPSS 22.0 software to conduct descriptive statistics, Pearson correlations, and independent-samples t-tests. There was no missing data in the study. The SPSS macro PROCESS (Model 6) developed by Hayes was employed to investigate the indirect effects of social capital on the relationship between personality and life satisfaction using 5000 bootstrapped replications [28]. Only mediation analyses for the offline social capital facets were presented, because online social capital was not associated with well-being in our research. Direct and indirect effects with 95% confidence intervals (CIs) were estimated, and age and sex were controlled for. The effects are considered significant if the CI values do not include zero.

## Results

Table 1 presents the means, standard deviations, bivariate correlations, and composite reliability estimates. Bivariate correlations revealed that online bonding was positively associated with extraversion and negatively associated with neuroticism. Online bridging was positively associated with openness and negatively associated with conscientiousness. Online social capital was not associated with offline social capital or life satisfaction. Offline bonding was positively associated with extraversion, agreeableness, and openness. Offline bridging was positively associated with extraversion, agreeableness, conscientiousness, and openness and negatively associated with neuroticism. Both offline bonding and bridging were positively correlated with life satisfaction. For exact effect sizes, see Table 1.

Given that no significant relationship was found between online social capital and life satisfaction, our analysis focused on the mediating role of offline social capital in the association between personality traits and life satisfaction, particularly agreeableness and extraversion. We note that the agreeableness finding was not hypothesized, but the effect sizes were moderate; therefore, these findings are presented in the main body of this work, and the direct and indirect effects of these two personality traits on life satisfaction as well as their indirect effects through offline bridging and bonding are presented in Tables 2 and 3. Effects of the remaining personality traits are shown in the Supplementary Material (see Supplementary Material 1).

**Fig. 1:**
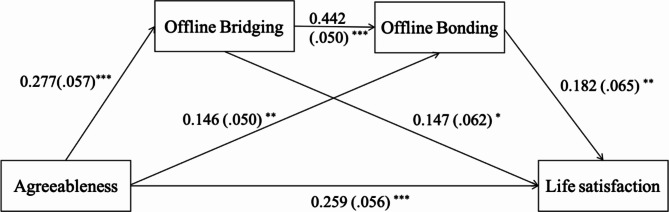
Path model of agreeableness on life satisfaction. The numbers reflect standardized path coefficients

Agreeableness had a direct effect on life satisfaction (β = 0.259, SE = 0.056, *p* < .001, 95% CI [0.150, 0.369]). The indirect effect of agreeableness on life satisfaction through offline bridging was significant (β = 0.041, SE = 0.021, 95% CI [0.003, 0.087]), as was the indirect pathway through offline bonding (β = 0.027, SE = 0.014, 95% CI [0.004, 0.058]). Furthermore, the chained mediation pathway, which proceeded from offline bridging to offline bonding, was also significant (β = 0.022, SE = 0.010, 95% CI [0.005, 0.045]), see Fig. 1 and Table 2.

**Fig. 2:**
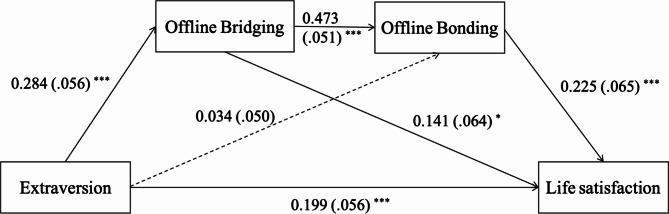
Path model of extraversion on life satisfaction. The numbers reflect standardized path coefficients

Extraversion had a direct effect on life satisfaction (β = 0.199, SE = 0.056, *p* < .001, 95% CI [0.090, 0.308]). While the indirect effect of offline bridging was significant (β = 0.040, SE = 0.022, 95% CI [0.006, 0.088]), the indirect effect of offline bonding was not (β = 0.008, SE = 0.012, 95% CI [-0.014, 0.035]). Nevertheless, the indirect pathway through offline bridging to offline bonding was significant (β = 0.030, SE = 0.012, 95% CI [0.010, 0.057]); see Fig. 2 and see Table 3.

## Discussion

The aim of the study was to explore the associations among the Big Five Personality traits, social capital facets (bonding and bridging) in online and offline contexts, and life satisfaction. This research question is of relevance, because people use platforms such as social media these days to reach out to others and establish social capital (at the moment of writing more than five billion people use a social media platform) [29]. It is well known that social capital can be a source of well-being (see this review [30]). Against this background the question arises how established personality links with well-being (e.g. extraversion and life satisfaction; [17]) might be mediated by both offline vs. online social capital social capital. Interestingly, our present findings revealed that offline social capital in the form of bridging and bonding played a significant role in mediating the relationship between both agreeableness and extraversion with life satisfaction. No significant association was observed between online social capital and life satisfaction.

Our results confirm the significant role of extraversion and agreeableness in offline bonding and bridging social capital. In line with previous research [13, 20], extraverted (and to lesser extent agreeable [14]) individuals tended to have higher levels of bonding and bridging social capital. Extraverted individuals, being sociable and outgoing, might naturally develop larger social networks, whereas agreeable individuals, being trustworthy and cooperative [31], effectively nurture their relationships. These characteristics contribute to the establishment and maintenance of social capital.

The results showed that extraverted and agreeable individuals may experience higher life satisfaction because of their personality traits and the social capital they accumulate and utilize. The variations in the mediation results (see also results from the supplementary material with the remaining personality traits) may be due to the distinct ways in which these personality traits influence social interactions. Agreeable individuals tend to be generally cooperative, compassionate, and more likely to form strong, supportive connections within their networks, contributing to both bridging and bonding social capital [32]. By contrast, extraverted individuals tend to be more outgoing, sociable, and prone to engaging with a broader range of people, which may facilitate the formation of bridging social capital more than bonding [13].

Interestingly, the association between personality traits and social capital was weaker in the online context than in the offline context. This finding could be due to limitations in communication and social interactions on online platforms, which may not fully facilitate the development and utilization of social capital [33]. However, as the digital landscape continues to evolve, future research should examine the influences of emerging technologies and platforms on these relationships.

The lack of a significant association between online social capital and life satisfaction raises questions about the value of online social networks in promoting well-being, particularly in an increasingly digitalized world. This finding is in line with that of a previous study [34], which can be explained by Maslow’s theory [21], suggesting that direct social interactions, as indicated by offline bridging and bonding, are crucial for well-being.

Despite these insights, our study had several limitations. The cross-sectional design precludes the establishment of causality, and longitudinal studies can offer more robust evidence. Additionally, self-report measures may be subject to response bias, highlighting the need for objective measures or alternative data sources in future research. In this brief report, we present further associations in the Supplementary Material section. As these tests have not been hypothesized and are exploratory, we do not discuss them at this point but hope that these findings encourage other scientists to seek replication (models touching upon the personality traits of conscientiousness, neuroticism, and openness). Please note that the agreeableness findings were also not hypothesized and therefore need to be replicated. Finally, this work focused on personality and social capital/well-being. Therefore, other factors should also be investigated in the future. For instance, a recent study showed that the perceived quality of WeChat (seen as a product) was linked to greater user belongingness (perhaps resulting in more social capital) [35]. Also social media usage frequency might be important in understanding levels of online social capital. In line with this finding, recent work among others established links between WeChat usage frequency to levels of interacting with people on the platform (and also to trust other WeChat users) [36]. These dimensions might be interesting additions to the variables investigated in the present work.

In conclusion, this study offers valuable insights into the connections between personality traits, social capital, and well-being. The findings emphasize the importance of extraversion and agreeableness in fostering offline social capital, and underscore the role of offline social capital in promoting life satisfaction. These results suggest that nurturing and maintaining offline social relationships are crucial for overall well-being. This nurturing/maintenance might also reflect our evolutionary heritage with having a need for direct social interactions [21, 37]. We believe that online social capital in particular can be valuable, when it also results in greater offline social capital. This is something, which future studies could focus on, also in the context of personality psychology.

**Table 1:** Descriptive statistics and correlation matrix

	1	2	3	4	5	6	7	8	9	10	Male (***M*** ± *SD*)	Female (***M*** ± *SD*)		*t*	*df*	***p***
1. Extraversion	1										25.52 ± 6.83	25.24 ± 6.81		0.308	287	0.758
2. Agreeableness	0.064	1									35.01 ± 5.09	35.89 ± 5.90		-1.138	287	0.256
3. Conscientiousness	0.220^**^	0.088	1								30.22 ± 6.11	31.81 ± 5.69		-2.022	287	0.044
4. Neuroticism	− 0.358^**^	− 0.221^**^	− 0.218^**^	1							23.05 ± 5.64	25.85 ± 6.61		-3.234	287	0.001
5. Openness	0.232^**^	0.087	0.038	− 0.068	1						34.81 ± 6.78	34.40 ± 6.71		0.445	287	0.657
6. Online bonding	0.161^**^	− 0.034	− 0.026	− 0.119^*^	0.113	1					24.67 ± 7.39	23.51 ± 8.17		1.075	287	0.283
7. Online bridging	0.027	0.027	− 0.170^**^	− 0.044	0.149^*^	0.498^**^	1				30.45 ± 7.94	29.25 ± 8.45		1.071	287	0.285
8. Offline bonding	0.156^**^	0.305^**^	0.105	− 0.062	0.120^*^	− 0.076	0.017	1			38.04 ± 7.15	40.21 ± 6.65		-2.367	287	0.019
9. Offline bridging	0.278^**^	0.290^**^	0.144^*^	− 0.184^**^	0.203^**^	− 0.021	0.055	0.521^**^	1		33.92 ± 6.25	34.97 ± 7.15		-1.123	287	0.262
10. Life satisfaction	0.269^**^	0.366^**^	0.261^**^	− 0.469^**^	0.115	0.044	0.016	0.365^**^	0.329^**^	1	20.99 ± 6.70	21.75 ± 6.86		-0.822	287	0.412
Cronbach α	0.878	0.758	0.818	0.854	0.804	0.871	0.893	0.864	0.868	0.891						
Mean	25.308	35.671	31.405	25.142	34.505	23.803	29.550	39.664	34.706	21.554						
*SD*	6.803	5.714	5.827	6.482	6.719	7.988	8.327	6.832	6.936	6.814						

** *p* < 0.01

* *p* < 0.05

**Table 2:** Direct and indirect effects of agreeableness on life satisfaction

		β	SE	LCI	UCI
Total effect		0.349	0.055	0.241	0.456
Direct effect		0.259	0.056	0.150	0.369
Indirect effects					
Total indirect effects		0.090	0.026	0.043	0.144
Agreeableness -> Offline bridging -> Life satisfaction		0.041	0.021	0.003	0.087
Agreeableness -> Offline bonding -> Life satisfaction		0.027	0.014	0.004	0.058
Agreeableness -> Offline bridging -> Offline bonding -> Life satisfaction		0.022	0.010	0.005	0.045

*Notes.* LCI = lower confidence interval; UCI = upper confidence interval. 95% confidence intervals were used. Gender and age were controlled for in the mediation analysis

**Table 3:** Direct and indirect effects of extraversion on life satisfaction

	β	SE	LCI	UCI
Total effect	0.277	0.056	0.167	0.387
Direct effect	0.199	0.056	0.090	0.308
Indirect effects				
Total indirect effects	0.078	0.027	0.030	0.137
Extraversion -> Offline bridging -> Life satisfaction	0.040	0.022	0.006	0.088
Extraversion -> Offline bonding -> Life satisfaction	0.008	0.012	− 0.014	0.035
Extraversion -> Offline bridging -> Offline bonding -> Life satisfaction	0.030	0.012	0.010	0.057

*Notes.* LCI = lower confidence interval; UCI = upper confidence interval. 95% confidence intervals were used. Gender and age were controlled for in the mediation analysis

## Electronic supplementary material

Below is the link to the electronic supplementary material.

Supplementary Material 1

## Data Availability

The data will be made available upon reasonable request.
