# Publisher Correction: Personality associations with online vs. offline social capital and well-being variables

**DOI:** 10.1186/s40359-025-02368-x

**Published:** 2025-01-29

**Authors:** Miao Chao, Dmitri Rozgonjuk, Jon D. Elhai, Haibo Yang, Christian Montag

**Affiliations:** 1 Key Research Base of Humanities and Social Sciences of the Ministry of Education,, Academy of Psychology and Behavior, Tianjin Normal University, Tianjin, China; 2 Department of Molecular Psychology, Institute of Psychology and Education, Ulm University, Helmholtzstr. 8/1, Ulm, 89081 Germany; 3 Institute of Computer Science, University of Tartu, Tartu, Estonia; 4 Department of Psychology, and Department of Psychiatry, University of Toledo, Toledo, OH USA; 5 Faculty of Psychology, Tianjin Normal University, Tianjin, China

**Correction: BMC Psychol 12, 763 (2024)**

**https://doi.org/10.1186/s40359-024-02265-9**

Following publication of the original article, it came to the journal's attention that as a result of a technical error, miscellaneous corrections provided by the authors during proofing of the article were not implemented. Please be referred to Supplementary Material 1, which details the miscellaneous corrections in question. The corrections detailed in this file have now been implemented in the original article. The publisher thanks you for reading this erratum and apologizes for any inconvenience caused.

## Supplementary Information

Supplementary Material 1. Miscellaneous post-publication corrections.
