# Supplementary material for: Personality associations with online vs. offline social capital and life satisfaction
Source: BMC Psychol. 2024 Dec 19;12:763. doi: 10.1186/s40359-024-02265-9 (PMC11657760; doi:10.1186/s40359-024-02265-9)
Supplement: Supplementary file 1 — Supplementary Material 1 [file 40359_2024_2265_MOESM1_ESM.docx]

**Supplementary material**

| S-Table 1. Direct and indirect effects of conscientiousness on life satisfaction. | |  |  |  |
| --- | --- | --- | --- | --- |
|  | β | SE | LCI | UCI |
| Total effect | .271 | .056 | .160 | .381 |
| Direct effect | .222 | .054 | .116 | .328 |
| Indirect effects |  |  |  |  |
| Total indirect effects | .048 | .022 | .006 | .096 |
| Conscientiousness -> Offline bridging -> Life satisfaction | .024 | .015 | .001 | .059 |
| Conscientiousness -> Offline bonding -> Life satisfaction | .008 | .011 | -.013 | .031 |
| Conscientiousness -> Offline bridging -> Offline bonding -> Life satisfaction | .016 | .009 | .001 | .036 |

*Notes.* LCI = lower confidence interval; UCI = upper confidence interval. 95% confidence intervals were used. Gender and age were controlled for in the mediation analysis.

|  |  |  |  |
| --- | --- | --- | --- |


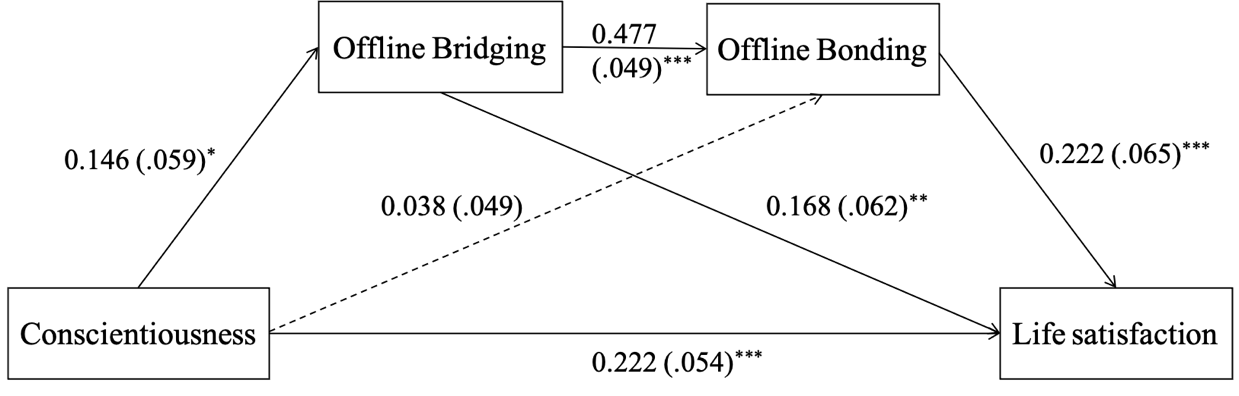


S-Figure 1. *Path model of conscientiousness on life satisfaction*

*The numbers reflect standardized path coefficients.*

| S-Table 2. Direct and indirect effects of neuroticism on life satisfaction. | |  |  |  |
| --- | --- | --- | --- | --- |
|  | β | SE | LCI | UCI |
| Total effect | -.522 | .051 | -.622 | -.422 |
| Direct effect | -.472 | .050 | -.570 | -.375 |
| Indirect effects |  |  |  |  |
| Total indirect effects | -.050 | .020 | -.092 | -.012 |
| Neuroticism -> Offline bridging -> Life satisfaction | -.022 | .015 | -.057 | .004 |
| Neuroticism -> Offline bonding -> Life satisfaction | -.004 | .011 | -.027 | .019 |
| Neuroticism -> Offline bridging -> Offline bonding -> Life satisfaction | -.023 | .001 | -.045 | -.007 |

*Notes.* LCI = lower confidence interval; UCI = upper confidence interval. 95% confidence intervals were used. Gender and age were controlled for in the mediation analysis.

| ﻿ |  |  |  |
| --- | --- | --- | --- |


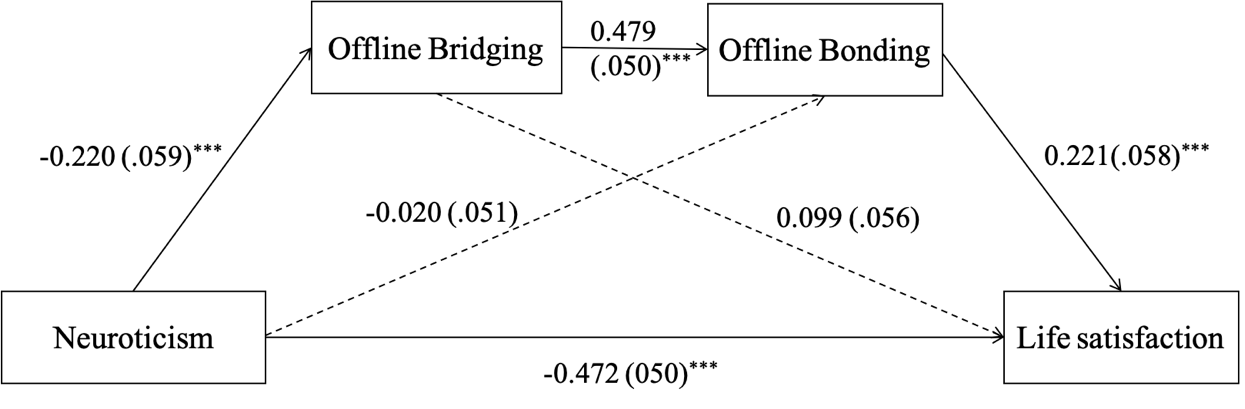


S-Figure 2. *Path model of neuroticism on life satisfaction*

*The numbers reflect standardized path coefficients.*

| S-Table 3. Direct and indirect effects of openness on life satisfaction. | |  |  |  |
| --- | --- | --- | --- | --- |
|  | β | SE | LCI | UCI |
| Total effect | .117 | .058 | .003 | .230 |
| Direct effect | .050 | .055 | -.059 | .160 |
| Indirect effects |  |  |  |  |
| Total indirect effects | .066 | .025 | .022 | .119 |
| Openness -> Offline bridging -> Life satisfaction | .038 | .018 | .008 | .079 |
| Openness -> Offline bonding -> Life satisfaction | .006 | .011 | -.015 | .030 |
| Openness -> Offline bridging -> Offline bonding -> Life satisfaction | .023 | .011 | .005 | .046 |
| *Notes.* LCI = lower confidence interval; UCI = upper confidence interval. 95% confidence intervals were used. Gender and age were controlled for in the mediation analysis. |  |  |  |  |


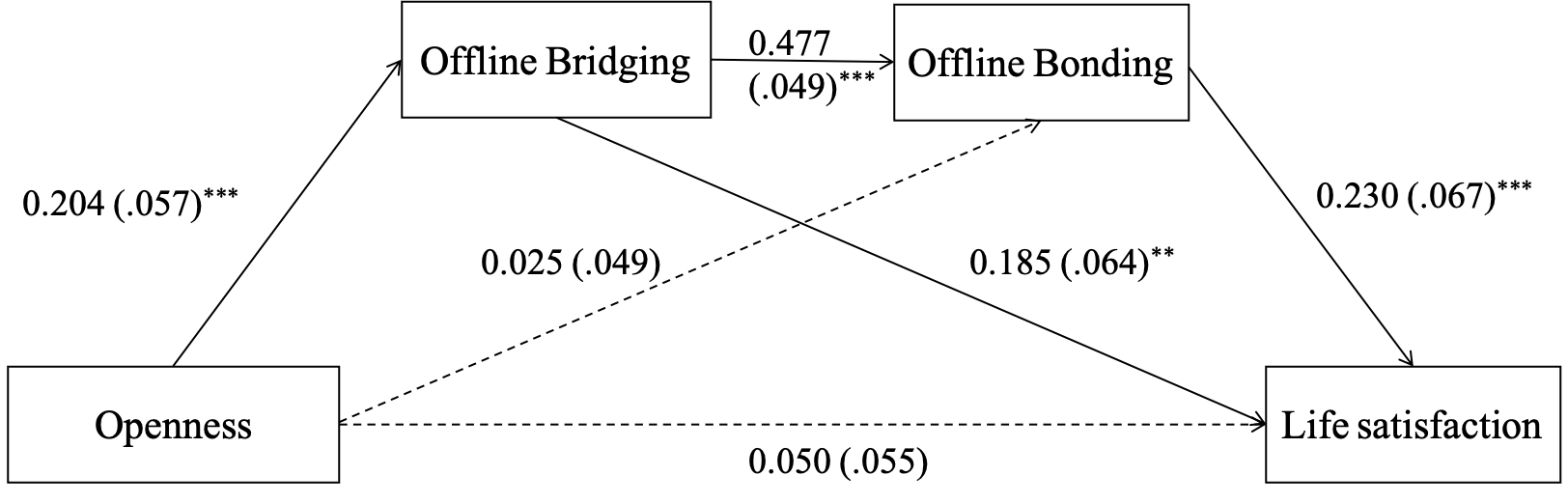


S-Figure 3. *Path model of openness on life satisfaction*

*The numbers reflect standardized path coefficients.*
